# Supplementary material for: GtcA is required for LTA glycosylation in Listeria monocytogenes serovar 1/2a and Bacillus subtilis
Source: Cell Surf. 2020 Feb 19;6:100038. doi: 10.1016/j.tcsw.2020.100038 (PMC7389260; doi:10.1016/j.tcsw.2020.100038)
Supplement: Supplementary data 1 [file mmc1.pdf]

SUPPLEMENTAL MATERIAL

SUPPLEMENTAL TABLES

Table S1: Bacterial strains used in this study

Unique ID	Strain name and resistance	Source
***Escherichia coli* strains**		
ANG201	*E. coli* pCN34; AmpR	(Charpentier et al., 2004)
ANG1264	DH5α pKSV7; AmpR	(Smith and Youngman, 1992)
ANG2223	XL1-Blue pKSV7-Δ*lmo2550*; AmpR	(Rismondo et al., 2018)
ANG2802	TG1 pDG1662; AmpR	(Guerout-Fleury et al., 1996)
ANG4243	XL1-Blue pIMK3; KanR	(Monk et al., 2008)
ANG4738	XL1-Blue pKSV7-Δ*gtlB*; AmpR	(Rismondo et al., 2018)
ANG4911	XL1-Blue pKSV7-Δ*gtcA*; AmpR	This study
ANG5026	XL1-Blue pIMK3-*gtcA*; KanR	This study
ANG5093	XL1-Blue pD1662-P_{ywcC}-*ywcC/gtcA*; AmpR	This study
ANG5182	XL1-Blue pIMK3-*csbB/yfhO*; KanR	This study
ANG5619	XL1-Blue pKSV7-Δ*lmo0215*; AmpR	This study
ANG5620	XL1-Blue pIMK3-$gtcA_{A65S}$; KanR	This study
ANG5621	XL1-Blue pIMK3-$gtcA_{N69A}$; KanR	This study
ANG5622	XL1-Blue pIMK3-$gtcA_{V73A}$; KanR	This study
ANG5623	XL1-Blue pIMK3-$gtcA_{F74A}$; KanR	This study
ANG5624	XL1-Blue pIMK3-$gtcA_{F91A}$; KanR	This study
ANG5625	XL1-Blue pIMK3-$gtcA_{R95A}$; KanR	This study
ANG5626	XL1-Blue pIMK3-$gtcA_{K121A}$; KanR	This study
ANG5627	XL1-Blue pIMK3-$gtcA_{N132A}$; KanR	This study
ANG5628	XL1-Blue pIMK3-*his-gtcA*; KanR	This study
ANG5629	XL1-Blue pIMK3-*his*-$gtcA_{A65S}$; KanR	This study
ANG5630	XL1-Blue pIMK3-*his*-$gtcA_{N69A}$; KanR	This study
ANG5631	XL1-Blue pIMK3-*his*-$gtcA_{V73A}$; KanR	This study
ANG5632	XL1-Blue pIMK3-*his*-$gtcA_{F74A}$; KanR	This study
ANG5633	XL1-Blue pIMK3-*his*-$gtcA_{F91A}$; KanR	This study
ANG5634	XL1-Blue pIMK3-*his*-$gtcA_{R95A}$; KanR	This study
ANG5635	XL1-Blue pIMK3-*his*-$gtcA_{K121A}$; KanR	This study
ANG5636	XL1-Blue pIMK3-*his*-$gtcA_{N132A}$; KanR	This study
***Bacillus subtilis* strains**		This study
ANG1691	168; trpC2	(Burkholder and Giles, 1947)
ANG2749	168Δ*csbB::kan*; KanR	(Rismondo et al., 2018)
ANG5047	168Δ*gtcA::kan*; KanR	This study
ANG5102	168Δ*gtcA::kan amyE::*P_{ywcC}-*ywcC/gtcA*; KanR CamR	This study
***Listeria monocytogenes* strains**		
ANG1263	10403S; serovar 1/2a; StrepR	(Bishop and Hinrichs, 1987)
ANG2325	10403SΔ*gtlA*; StrepR	(Percy et al., 2016)
ANG2794	10403SΔ*lmo1079*; StrepR	(Rismondo et al., 2018)

ANG4264	10403SΔ*gtlB*; StrepR	(Rismondo et al., 2018)
ANG4972	10403SΔ*gtcA*; StrepR	This study
ANG5031	10403SΔ*gtcA* pIMK3-*gtcA*; StrepR KanR	This study
ANG5195	10403SΔ*gtlA*Δ*gtlB*; StrepR	This study
ANG5197	10403SΔ*lmo1079*Δ*lmo2550*; StrepR	This study
ANG5199	10403SΔ*gtlA*Δ*gtlB* pIMK3-*csbB/yfhO*; StrepR KanR	This study
ANG5203	10403SΔ*lmo1079*Δ*lmo2550* pIMK3-*csbB/yfhO*; StrepR KanR	This study
ANG5638	10403SΔ*lmo0215*; StrepR	This study
ANG5639	10403SΔ*gtcA* pIMK3-$gtcA_{A65S}$; StrepR KanR	This study
ANG5640	10403SΔ*gtcA* pIMK3-$gtcA_{N69A}$; StrepR KanR	This study
ANG5641	10403SΔ*gtcA* pIMK3-$gtcA_{V73A}$; StrepR KanR	This study
ANG5642	10403SΔ*gtcA* pIMK3-$gtcA_{F74A}$; StrepR KanR	This study
ANG5643	10403SΔ*gtcA* pIMK3-$gtcA_{F91A}$; StrepR KanR	This study
ANG5644	10403SΔ*gtcA* pIMK3-$gtcA_{R95A}$; StrepR KanR	This study
ANG5645	10403SΔ*gtcA* pIMK3-$gtcA_{K121A}$; StrepR KanR	This study
ANG5646	10403SΔ*gtcA* pIMK3-$gtcA_{N132A}$; StrepR KanR	This study
ANG5647	10403SΔ*gtcA* pIMK3-*his-gtcA*; StrepR KanR	This study
ANG5648	10403SΔ*gtcA* pIMK3-*his*-$gtcA_{A65S}$; StrepR KanR	This study
ANG5649	10403SΔ*gtcA* pIMK3-*his*-$gtcA_{N69A}$; StrepR KanR	This study
ANG5650	10403SΔ*gtcA* pIMK3-*his*-$gtcA_{V73A}$; StrepR KanR	This study
ANG5651	10403SΔ*gtcA* pIMK3-*his*-$gtcA_{F74A}$; StrepR KanR	This study
ANG5652	10403SΔ*gtcA* pIMK3-*his*-$gtcA_{F91A}$; StrepR KanR	This study
ANG5653	10403SΔ*gtcA* pIMK3-*his*-$gtcA_{R95A}$; StrepR KanR	This study
ANG5654	10403SΔ*gtcA* pIMK3-*his*-$gtcA_{K121A}$; StrepR KanR	This study
ANG5655	10403SΔ*gtcA* pIMK3-*his*-$gtcA_{N132A}$; StrepR KanR	This study

Table S2: Primers used in this study

Number	Name	Sequence
ANG2979	gtcA up fw2	GCGCGGATCCGGTAGTAAGGATAAGACACTGG
ANG2980	gtcA up rev2	CAAGCAAGATTAGTTCATACTATGTCTTCTTTCTCTC
ANG2981	gtcA down fw2	CATAGTATGAACTAATCTTGCTTGTTTGCTTCAAC
ANG2982	gtcA down rev2	GCGCGGTACCGATCATCCAGTACATCAGACGG
ANG3036	gtcA pIMK3 fw NcoI	CATGCCATGGGGAACAAAATAAGAAAATGGTTAGACA AG
ANG3037	gtcA pIMK3 rev SalI	GCGCGTCGACTTATTTTTCACTTTGAAAATGATCC
ANG3068	fw 1kb gtcA	CACATAGTTGCTCGGTGTTC
ANG3069	Front ApaI gtcA	CGCGGGCCCGGTTGTGAAAACCCCCATAATG
ANG3070	Back XhoI gtcA	GCCGCTCGAGTTTAAAAAAACAAAAGAAGAAGGGG
ANG3071	rev 1 kb gtcA	GCCGTCTTAATAGCACGATG
ANG3089	5-BamHI-ywcC with P	CGCGGATCCAGTTTTCATAGTAACCTCCAATTG
ANG3090	3-HindIII-gtcA BSU	CCCAAGCTTGCTGCAGTCCTCTCATACC
ANG3143	pIMK3-*csbB/yfhO* fw	GCGCCCATGGGGAAGCAAGGATTAATCTCG
ANG3144	pIMK3-*csbB/yfhO* rev	GCGCGTCGACCTATATAGAGCCGGGCTTTTTAC
ANG3215	gtcA A65S fw	CGTCCTTTTTTCATATTTTTCTAATAAAAAATATGTTTTT G
ANG3216	gtcA A65S rev	TAGAAAAATATGAAAAAAGGACGGATGCAACC
ANG3217	gtcA N69A fw	CATATTTTTCTGCTAAAAAATATGTTTTTGAAAGCTATA C
ANG3218	gtcA N69A rev	CATATTTTTTAGCAGAAAAATATGCAAAAAGGACGG
ANG3219	gtcA V73A fw	TAAAAAATATGCTTTTGAAAGCTATACACCTACTTG
ANG3220	gtcA V73A rev	AGCTTTCAAAAGCATATTTTTTATTAGAAAAATATGCA AAAAG
ANG3221	gtcA F74A fw	AAAATATGTTGCTGAAAGCTATACACCTACTTGG
ANG3222	gtcA F74A rev	GTATAGCTTTCAGCAACATATTTTTTATTAGAAAAATAT GCAA
ANG3223	gtcA F91A fw	GTTACATCAGCTTTCGGTTTCCGCTTTTTGAC
ANG3224	gtcA F91A rev	GGAAACCGAAAGCTGATGTAACTTCTCGAGCGC
ANG3225	gtcA R95A fw	TTTCGGTTTCGCCTTTTTGACTTATTTAGTGGACATTT
ANG3226	gtcA R95A rev	AGTCAAAAAGGCGAAACCGAAAAATGATGTAACTTC
ANG3227	gtcA K121A fw	ATTATGGGCAGCAATTTGGACGAATGTAATTGTCC
ANG3228	gtcA K121A rev	TCGTCCAAATTGCTGCCCATAATTCATTTATAGATAAAA
ANG3229	gtcA N132A fw	CCTTGTACTTGCTTATGTGTTTAGTAAATGGATCATTT
ANG3230	gtcA N132A rev	CTAAACACATAAGCAAGTACAAGGACAATTACATTCG
ANG3305	lmo0215 up fw BamHI	CGCGGATCCCTGCGATGGTTCCTGATG
ANG3306	lmo0215 up rev	TAATTTATATCTCATTAGTCGCTTCATGGAACGTTCTGA CAC
ANG3307	lmo0215 down fw	AAGCGACTAATGAGATATAAATTACTCGGTCCAAAAGA TTTTG
ANG3308	lmo0215 down rev KpnI	CGGGGTACCCTCGGCTCTGACTTACTC
ANG3345	His-*gtcA* fw 2	CACCACCACCACAACAAAATAAGAAAATGGTTAGAC
ANG3346	NcoI-His 2	CATGCCATGGGGCACCACCACCACCACCACAACAAA

Table S3: Importance of conserved GtcA amino acid residues for the glycosylation of WTA and LTA in *L. monocytogenes*

	Producing GtcA		Producing His-GtcA	
Strain	WTA	LTA	WTA	LTA
Δ*gtcA*+*gtcA*	+++	+++	+++	+++
Δ*gtcA*+$gtcA_{A65S}$	+++	+++	+++	+++
Δ*gtcA*+$gtcA_{N69A}$	+++	+++	+++	+++
Δ*gtcA*+$gtcA_{V73A}$	+++	+++	+++	+++
Δ*gtcA*+$gtcA_{F74A}$	+/-	+++	-	+
Δ*gtcA*+$gtcA_{F91A}$	+	+++	+/-	+++
Δ*gtcA*+$gtcA_{R95A}$	-	-	-	-
Δ*gtcA*+$gtcA_{K121A}$	+++	-	+++	-
Δ*gtcA*+$gtcA_{N132A}$	-	-	-	-

+++ full complementation; +/- and + partial complementation; - no complementation

SUPPLEMENTAL FIGURES AND LEGENDS

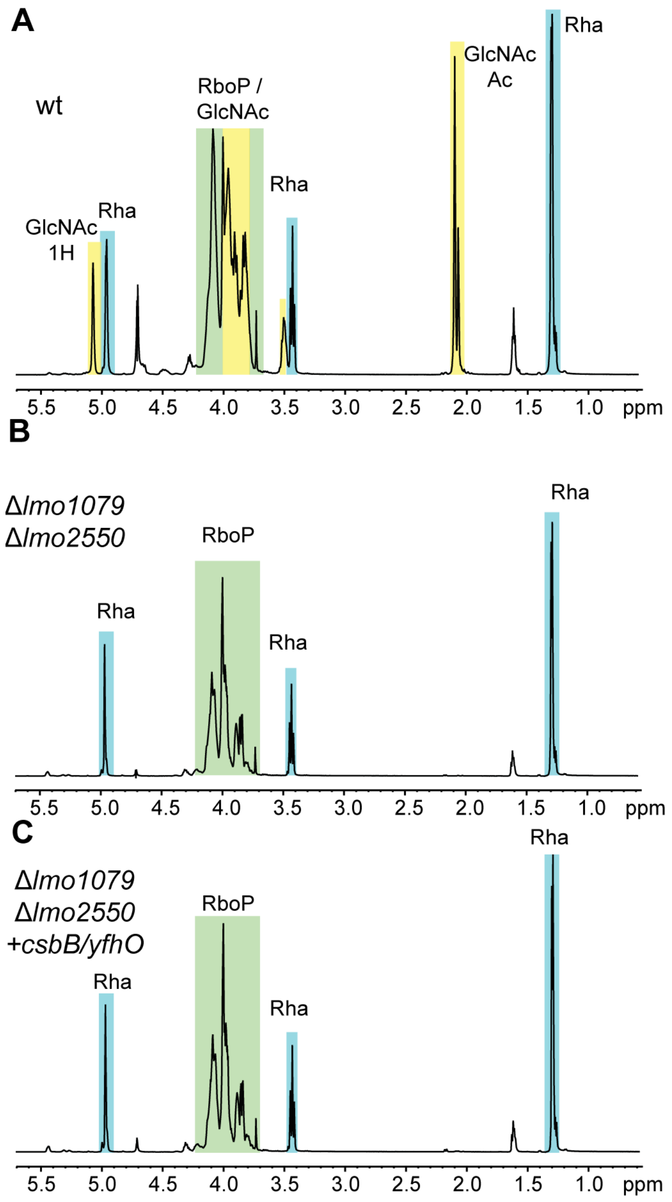

Figure S1: NMR analysis of WTA isolated from wildtype *L. monocytogenes* strain 10403S, the *lmo1079/lmo2550* mutant and the *lmo1079*/*lmo2550* mutant expressing the *B. subtilis csbB/yfhO* operon. (A-C) NMR spectra of WTA. WTA was extracted from *L. monocytogenes* (A) 10403S (wt), (B) the 10403SΔ*lmo1079*/Δ*lmo2550* mutant and (C) strain 10403SΔ*lmo1079* Δ*lmo2550*+*csbB/yfhO* (grown in presence of IPTG) and analyzed by NMR. Peaks of nonexchangeable protons were assigned to the different WTA components using previously published spectra and highlighted in colored boxes (Kamisango et al., 1983, Reichmann et al., 2013, Brauge et al., 2016). One representative result of two independent experiments is shown.

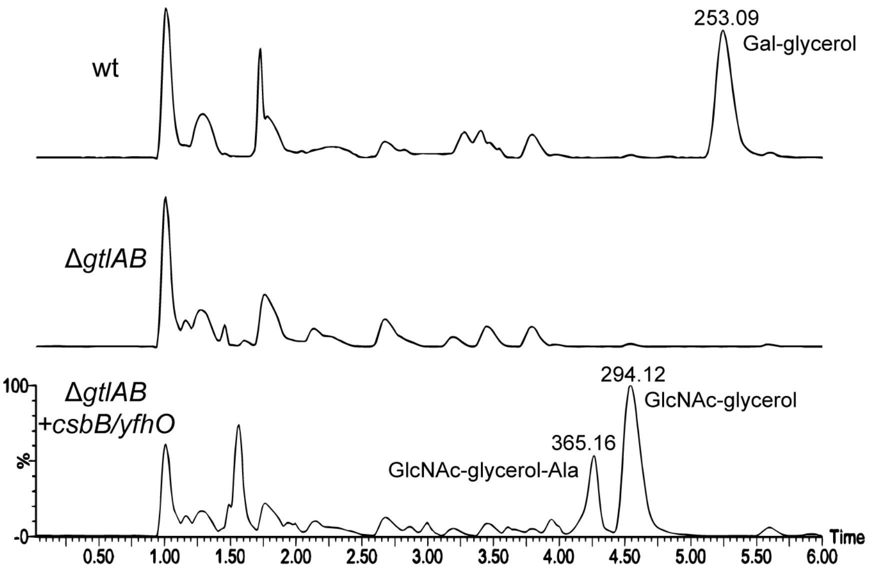

Figure S2: UPLC-MS/MS analysis of LTA fragments isolated from wildtype *L. monocytogenes* 10403S, the *gtlAB* mutant and the *gtlAB* mutant expressing the *B. subtilis csbB/yfhO* operon. Purified LTA was analyzed by UPLC-MS/MS as described in the methods section. Peaks corresponding to Gal-glycerol with a *m/z* of 253.09, GlcNAc-glycerol with a *m/z* of 294.12 and GlcNAc-glycerol-Ala with a *m/z* of 365.16 are labelled. The highest peak for the LTA sample extracted from strain 10403SΔ*gtlAB+csbB/yfhO* was set to 100%. One representative result of three independent experiments is shown.

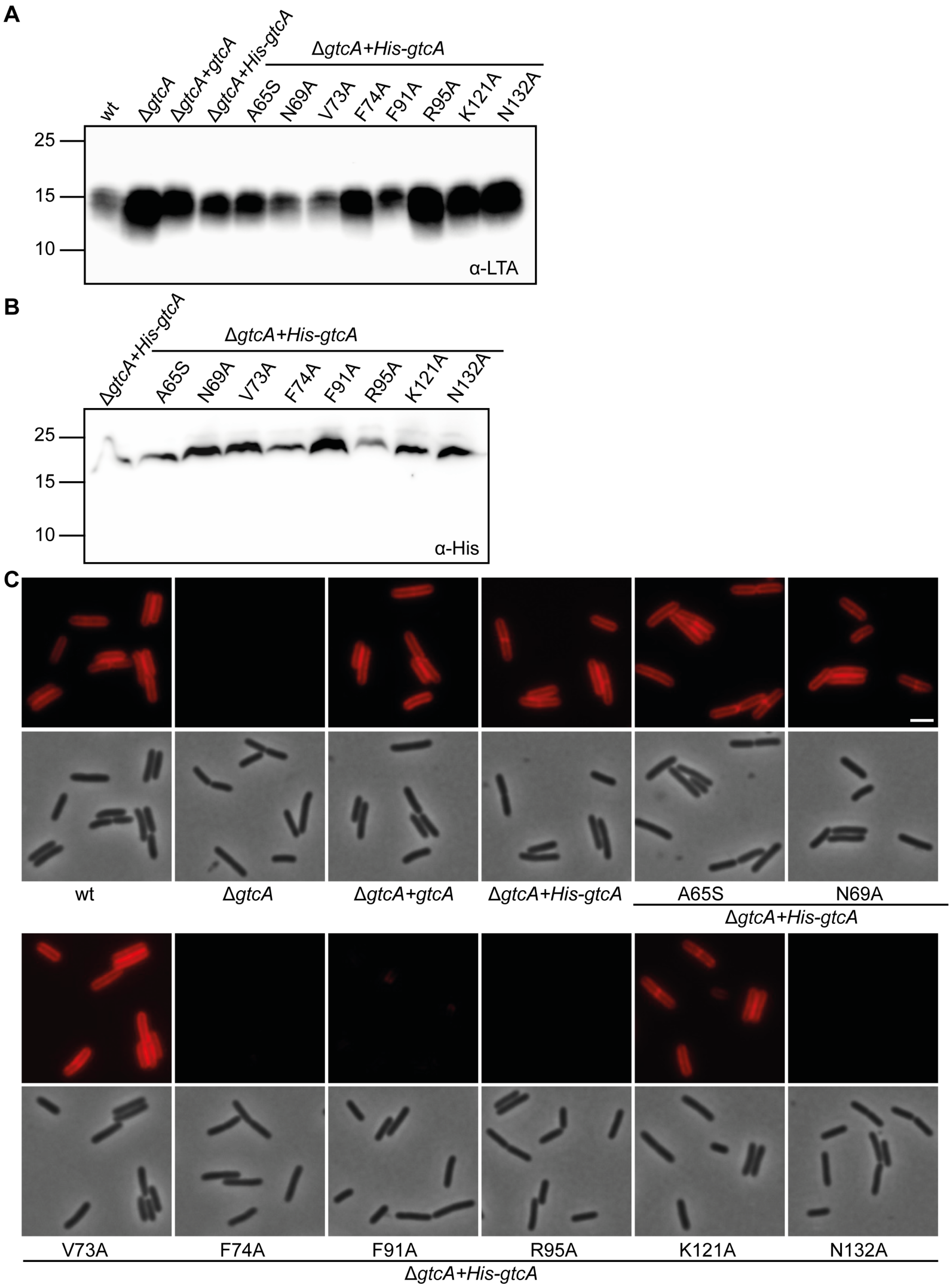

Figure S3: Identification of amino acid residues essential for the function and/or stability of His-tagged versions of the *L. monocytogenes* 10403S GtcA protein. (A) Detection of LTA by western blot. Cell extracts of the indicated *L. monocytogenes* strains expressing His-GtcA or derivatives thereof were prepared, separated on a 15% SDS PAGE and LTA detected using a polyglycerolphosphate-specific monoclonal antibody. Samples were standardised based on culture OD_{600} readings as described in the methods section. (B) Detection of His-GtcA proteins by western-blot. Protein extracts of the indicated *L. monocytogenes* strains were prepared as described in the method section and separated on a 15% Tricine

SDS PAGE gel. His-GtcA proteins were detected using a monoclonal poly-Histidine-Peroxidase antibody. (C) Microscopy analysis and detection of WTA glycosylation using the fluorescently labelled WGA-Alexa 594 lectin. Log-phase cells of the indicated *L. monocytogenes* strains were stained with WGA-Alexa 594 as described in the methods section and subjected to phase contrast and fluorescence microscopy. Scale bar is 2 μm. One representative result of three independent experiments is shown.

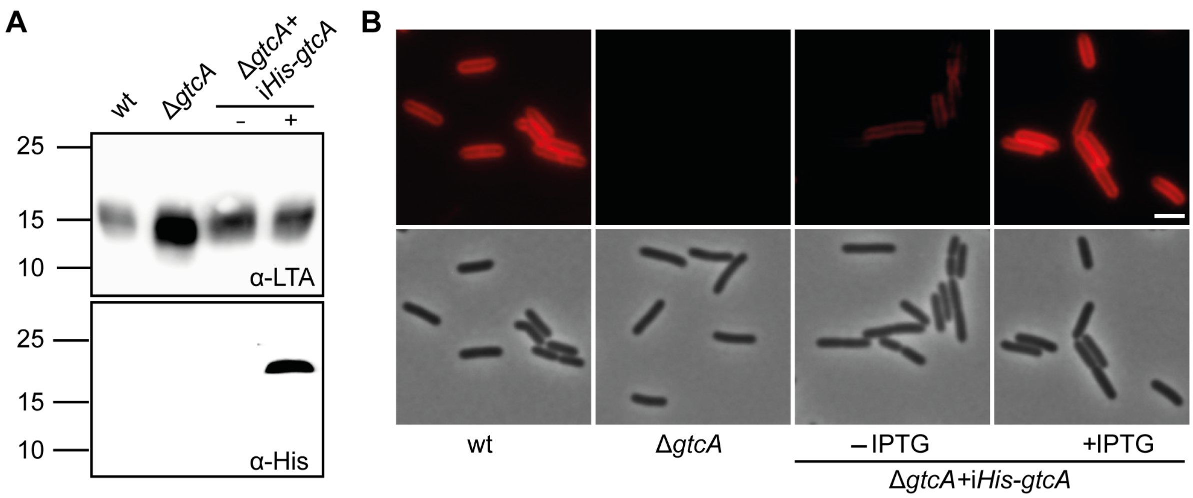

Figure S4: Complementation analysis using *L. monocytogenes* serovar 1/2a strain 10403SΔ*gtcA* expressing His-tagged GtcA. (A) Detection of LTA and His-GtcA by western blot. Cell and protein extracts of *L. monocytogenes* strains 10403S, 10403SΔ*gtcA* and strain 10403SΔ*gtcA* pIMK3-*His-gtcA* grown in the absence or presence of IPTG were prepared as described in the methods section. Upper panel: Cell extracts were loaded on a 15% SDS PAGE gel and LTA was detected by western blot using a polyglycerol-phosphate-specific monoclonal antibody. Lower panel: Protein extracts were loaded on 15% Tricine SDS PAGE gels and the His-GtcA protein were detected using a monoclonal polyHistidine-Peroxidase antibody. Samples were standardised based on culture OD_{600} readings as described in the methods section. (B) Microscopy analysis and detection of WTA glycosylation using the lectin WGA-Alexa 594. Log-phase cells of the indicated *L. monocytogenes* strains were stained with the WGA-Alexa 594 dye as described in the methods section and subjected to phase and fluorescence microscopy. Scale bar is 2 µm. One representative result of three independent experiments is shown.

REFERENCES

BISHOP, D. K. & HINRICHS, D. J. 1987. Adoptive transfer of immunity to *Listeria monocytogenes*. The influence of in vitro stimulation on lymphocyte subset requirements. *J Immunol,* 139**,** 2005-9.

BRAUGE, T., SADOVSKAYA, I., FAILLE, C., BENEZECH, T., MAES, E., GUERARDEL, Y. & MIDELET-BOURDIN, G. 2016. Teichoic acid is the major polysaccharide present in the *Listeria monocytogenes* biofilm matrix. *FEMS Microbiol Lett,* 363**,** fnv229.

BURKHOLDER, P. R. & GILES, N. H., JR. 1947. Induced biochemical mutations in *Bacillus subtilis*. *Am J Bot,* 34**,** 345-8.

CHARPENTIER, E., ANTON, A. I., BARRY, P., ALFONSO, B., FANG, Y. & NOVICK, R. P. 2004. Novel cassette-based shuttle vector system for gram-positive bacteria. *Appl Environ Microbiol,* 70**,** 6076-85.

GUEROUT-FLEURY, A. M., FRANDSEN, N. & STRAGIER, P. 1996. Plasmids for ectopic integration in *Bacillus subtilis*. *Gene,* 180**,** 57-61.

KAMISANGO, K., FUJII, H., OKUMURA, H., SAIKI, I., ARAKI, Y., YAMAMURA, Y. & AZUMA, I. 1983. Structural and immunochemical studies of teichoic acid of *Listeria monocytogenes*. *J Biochem,* 93**,** 1401-9.

MONK, I. R., GAHAN, C. G. & HILL, C. 2008. Tools for functional postgenomic analysis of *Listeria monocytogenes*. *Appl Environ Microbiol,* 74**,** 3921-34.

PERCY, M. G., KARINOU, E., WEBB, A. J. & GRÜNDLING, A. 2016. Identification of a Lipoteichoic Acid Glycosyltransferase Enzyme Reveals that GW-Domain-Containing Proteins Can Be Retained in the Cell Wall of *Listeria monocytogenes* in the Absence of Lipoteichoic Acid or Its Modifications. *J Bacteriol,* 198**,** 2029-42.

REICHMANN, N. T., CASSONA, C. P. & GRÜNDLING, A. 2013. Revised mechanism of D-alanine incorporation into cell wall polymers in Gram-positive bacteria. *Microbiology,* 159**,** 1868-77.

RISMONDO, J., PERCY, M. G. & GRÜNDLING, A. 2018. Discovery of genes required for lipoteichoic acid glycosylation predicts two distinct mechanisms for wall teichoic acid glycosylation. *J Biol Chem,* 293**,** 3293-3306.

SMITH, K. & YOUNGMAN, P. 1992. Use of a new integrational vector to investigate compartment-specific expression of the *Bacillus subtilis spoIIM* gene. *Biochimie,* 74**,** 705-11.
